# A first nation-wide assessment of soil-transmitted helminthiasis in Fijian primary schools, and factors associated with the infection, using a lymphatic filariasis transmission assessment survey as surveillance platform

**DOI:** 10.1371/journal.pntd.0008511

**Published:** 2020-09-25

**Authors:** Sung Hye Kim, J. Russell Stothard, Milika Rinamalo, Meleresita Rainima-Qaniuci, Nemani Talemaitoga, Mike Kama, Eric Rafai, Seoyun Jang, Ji Young Kim, Yoo Min Oh, Eun-Min Kim, Sung-Tae Hong, John H. Lowry, Jaco J. Verweij, Louise A. Kelly-Hope, Min-Ho Choi

**Affiliations:** 1 Department of Tropical Disease Biology, Liverpool School of Tropical Medicine, Liverpool, United Kingdom; 2 Ministry of Health, Dinem House, Suva, Republic of Fiji; 3 Department of Tropical Medicine and Parasitology and Institute of Endemic Diseases, Seoul National University College of Medicine, Seoul, Korea; 4 Department of Environmental Medical Biology and Arthropods of Medical Importance Resource Research Bank, Institute of Tropical Medicine, Yonsei University College of Medicine, Seoul, Korea; 5 School of Geography, Earth Science, and Environment, The University of South Pacific, Suva, Republic of Fiji; 6 Laboratory of Medical Microbiology and Immunology, Elisabeth Hospital, Tilburg, The Netherlands; University of New South Wales Faculty of Medicine, AUSTRALIA

## Abstract

**Background:**

Soil-transmitted helminthiasis (STH) is endemic in Fiji but its prevalence is not known and likely to have changed after a decade of mass drug administration (MDA) for lymphatic filariasis (LF). By linking with LF transmission assessment surveys (LF-TAS), we undertook the first nation-wide assessment of STH in Fijian primary schools, as well as an analysis of factors associated with STH infections.

**Methodology/Principal findings:**

A cross-sectional assessment for STH was conducted in all four Divisions of Fiji from 2014 to 2015. In the Western, Central, and Northern Divisions, schools were sub-sampled after LF-TAS, while, in the Eastern Division, schools were selected via simple random sampling. For the diagnosis of STH, stool samples were examined by coproscopy with a single Kato-Katz thick smear (KK) and the formol-ether-acetate concentration technique, except for the samples from the Eastern Division where only KK was used. Mean prevalence of any STH among class 1–2 students at the national level was 10.5% (95% CI: 6.9–15.5). Across the three Divisions via LF-TAS, the prevalence levels for ascariasis were 8.7% (95% CI: 4.3–16.6), hookworm 3.9% (95% CI: 2.3–6.6) and trichuriasis 0%. In the Eastern Division, ascariasis prevalence was 13.3% (95% CI: 6.4–25.6), and hookworm 0.7% (95% CI: 0.2–2.5), with one case of trichuriasis. Among class 3–8 students, ascariasis prevalence was lower. Lower risk of any STH was associated with wearing shoes (adjusted OR 0.54, 95% CI: 0.32–0.90) and having piped water from the Fiji Water Authority at home (adjusted OR 0.48, 95% CI: 0.25–0.92).

**Conclusions:**

After a decade of community-based LF-MDA, STH in school-age children in Fiji is now close to 10%, but localities of endemicity remain. Preventive chemotherapy should be maintained in areas with elevated STH prevalence alongside targeted delivery of integrated WASH interventions. LF-TAS has provided an opportunity to develop future public health surveillance platforms.

## Author summary

Soil-transmitted helminth infections (STH) are common in school-age children in the developing world and can cause morbidity to affected human beings depending on the intensity and duration of infection. In Fiji, where there has been a long history of implementing a program for eliminating lymphatic filariasis (LF) due to inadequate sanitation conditions, there is a need to assess the current epidemiological profile of STH since the LF mass drug administration (MDA) as a community-based intervention may soon stop. By linking with ongoing LF-transmission assessment surveys (LF-TAS), we undertook the first nation-wide assessment of STH in Fijian primary schools. Mean prevalence of STH across three Divisions of Fiji in class 1–2 students chosen for LF-TAS was 12.1% (ascariasis 8.7%, hookworm 3.9%, and trichuriasis 0%). In the remaining Division, the Eastern, ascariasis prevalence was 13.3%, and hookworm 0.7%. Wearing shoes and use of piped water reduced the prevalence of STH. It is concluded that STH in school-age children in Fiji is at low levels, but hot spot localities of elevated STH endemicity remain. Therefore, preventive chemotherapy should be maintained in these localities alongside targeted delivery of appropriate integrated water, sanitation and hygiene interventions.

## Introduction

Soil-transmitted helminthiasis (STH) is a global public health problem, typically in areas where poor sanitation and inadequate clean water abound, blighting childhood development [1]. The main species that infect people are the roundworm (*Ascaris lumbricoides*), the hookworms (*Necator americanus* and *Ancylostoma duodenale*), and the whipworm (*Trichuris trichiura*). According to WHO guidelines, the procedure of choice for controlling STH is to apply preventive chemotherapy (PCT), defined as the administration of medicines as a public-health tool to prevent selected neglected tropical diseases (NTDs). The frequency of treatment is directly linked to epidemiologic information on the infection, which needs to be updated to permit policy revisions [2]. To augment PCT, the provision of safe water, sanitation, and hygiene (WASH) is also encouraged, given that certain STH are transmitted via the fecal-oral routes.

Lymphatic filariasis (LF) shares with STH the common anthelminthic treatments, albendazole and ivermectin [3]. As such, an integrated approach within actions against neglected tropical diseases is strongly advocated by WHO [2]. In light of the above, up-to-date epidemiologic information on STH is needed, especially where LF mass drug administration (MDA) is down-scaling with the declining prevalence of LF. Under the Pacific Program to Eliminate Lymphatic Filariasis (PacELF), which recommended implementing community-based MDA with diethylcarbamazine citrate (DEC) 6 mg/kg and albendazole 400 mg once a year for children over two, countries have achieved effective MDA coverage and in most of them the LF program is on track to achieve the goal of elimination as a public health problem [4].

**Fig 1:**
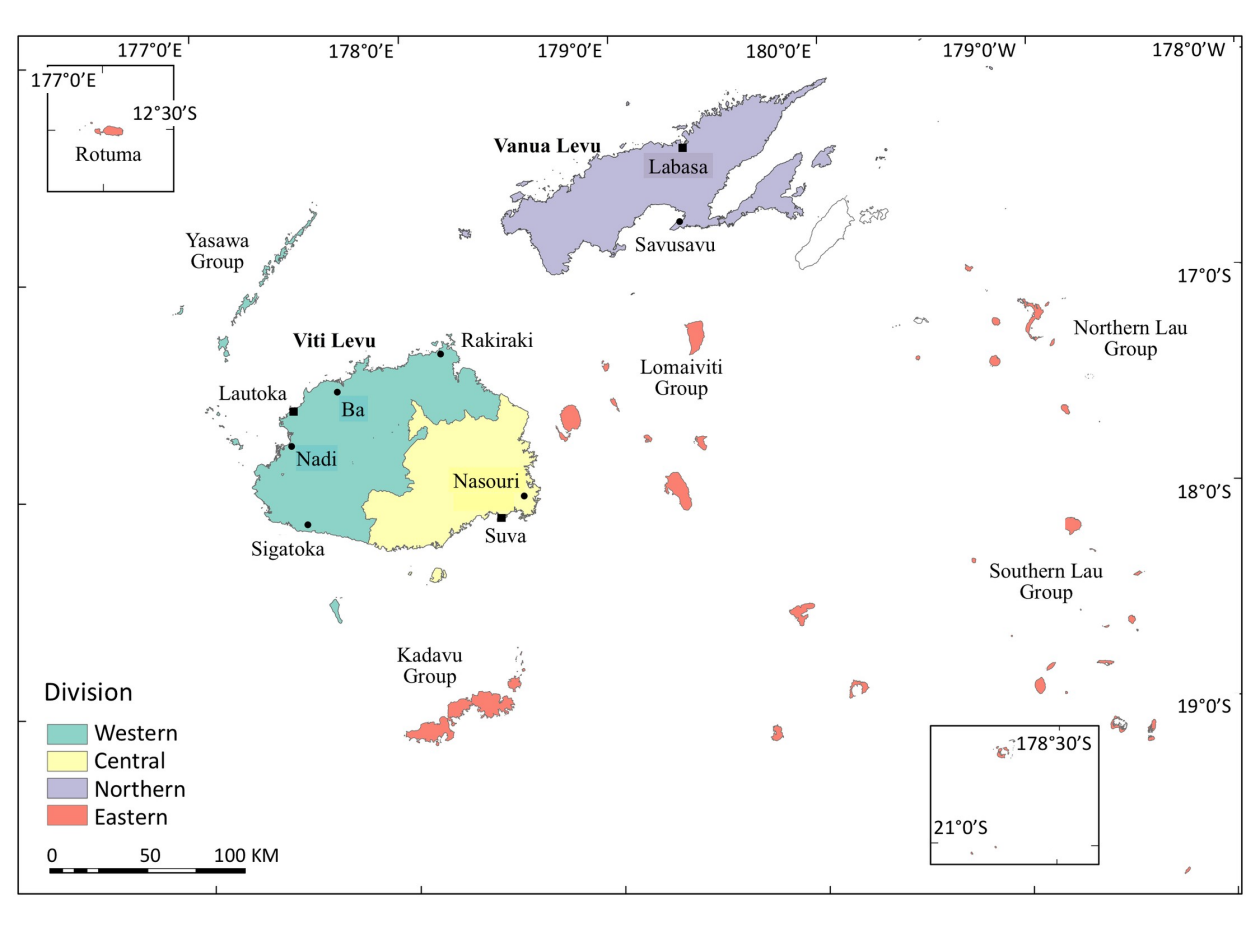
Sketch map of Fiji showing the 4 Divisions of Fiji covered by the STH prevalence assessment. The Northern Division here excludes the Taveuni sub-Division.

In Fiji, it was also possible to track back initial efforts to control STH when the annual round of community-based LF-MDA was begun in 2001 with the support of PacELF [5]. Most of the consecutive annual LF-MDA rounds were successful, with around 65% population coverage, and led to termination of LF-MDA decisions in three Divisions of the country [6,7]: the final LF-MDA 7^th^ round for the Western Division took place in 2009, and the 10^th^ for the Central and 9^th^ for the Northern in 2012. The Eastern Division islands and the Taveuni sub-Division have formed another LF implementation unit (Fig 1) for further LF-MDA rounds since 2013 [6]. The Fijian Ministry of Health and Medical Services (MHMS) also launched the National Iron and Micro-nutrient Supplementation (NIMS) program for school-age children (SAC), pre-SAC, women of childbearing age (WCBA), and lactating women in 2010. This was a five-year pilot project, based on the finding of highly prevalent anemia by the National Nutritional Survey conducted in 2007 [8], which became the background for six-monthly albendazole distributions together with iron supplementation for pre-SAC and SAC. The program coverage was only above 75% in its first year, and it did not achieve the global target equally among school-aged and preschool-aged children thereafter until 2014 [7].

Despite STH’s public health importance, and continued efforts to control it in Fiji, nationally representative data remain unavailable at the program level, which hampers both effective implementation and evaluation of current and future interventions [9]. For example, Bethani *et al*. reported in a study back in 1998 that prevalence in Fiji was 50% for hookworm, 11% for ascariasis and 2% for trichuriasis [10], whereas a multi-country survey conducted by Hughes *et al*. in 2000 reported a prevalence of 10% for any STH in a small sub-sample of urban school children [11]. Nevertheless, recent information on STH epidemiology is scarce, and like other countries in Oceania, Fiji is still classified as STH endemic, which requires further PCT by the WHO [3,12]. This may have been partly due to the absence of a contemporary epidemiologic profile to guide future PCT activities. Hence, a nation-wide cross-sectional STH prevalence assessment was planned in all four Divisions (Western, Central, Eastern, and Northern) of Fiji (Fig 1), in conjunction with LF transmission assessment surveys (LF-TAS) in the two main islands. The objective of the study was to obtain a mid-term epidemiologic profile of STH as a preliminary to developing a national strategy for STH control. Specifically, STH prevalence in each Division, the implementation unit for public health intervention, as well as at the national level, was to be estimated in order to define appropriate PCT frequencies, in case PCT is still needed.

Unlike for other NTDs, the approach for STH has been mainly considered “transmission control”, based on providing preventive chemotherapy as a public health intervention for all young children, preschool and school-age children living in areas where the baseline prevalence of any STH is 20% or higher among children, but there are problems in maintaining satisfactory progress with this strategy [12]. Meanwhile, elimination as a public health goal for STH with a moderate to heavy infection prevalence of less than 1% [13] is aligned with “morbidity control among children”. We assessed where the Fiji STH program was with regard to both intervention perspectives. Lastly, in addition to the parasitologic surveys, factors related to the infection, such as demographic and WASH-associated covariates were explored, considering that WASH interventions and their uptake could reduce STH transmission over time [14,15] and this would help MHMS in making evidence-based programmatic decisions for controlling STH.

## Methods

### Study area and survey timeframe

Fiji is an archipelago comprised of more than 330 islands lying between 12˚and 22˚S and 175˚ and 178˚E in the South Pacific [16]. Up to 835,000 Fijians reside on the 100 or so consistently- inhabited islands, with Viti Levu and Vanua Levu, the two major islands, populated by 87% of the total population [17]. There are two major ecological zones in the country: (1) Wet Zone, which receives rain > 3,000 mm/year; and (2) Dry Zone, which receives rain < 2,000 mm/year [16]. Most of the Western Division is Dry zone, which is further divided into Strong Dry Zone in the western half, and Moderate Dry in the eastern half, while the Central Division primarily is Wet Zone. The Northern Division is mostly Dry Zone in its northern aspect and Wet zone in the south [16]. Most of the Eastern Division belongs to Wet zone, except Rotuma Island located at the north of Viti Levu.

The STH prevalence assessment was conducted in a phased manner over the four Health Divisions of Fiji by having each Division as an evaluation unit (EU) (Fig 1). The survey in the Western Division was conducted at the beginning of 2014 in conjunction with the second LF-TAS (TAS 2) in the Division. Then the assessments were embedded with the TAS 1 for the Central Division in July-August 2014, and with the TAS 2 for the Northern Division in February-March 2015. For the Eastern Division, the survey was organized separately from LF-TAS, covering all five sub-Divisions of the Eastern Division in August 2015.

### Selection of target schools, and survey organization

For the three Divisions surveyed via LF-TAS, a detailed description of school selection procedures and the organization of the survey of the target population, namely class 1 and 2 students, has been presented elsewhere [5], with the summary of selected schools and sample sizes in Table 1. In the Eastern Division, 20 schools were first selected via simple random sampling from 4 sub-Divisions, and all 4 schools of the Rotuma sub-Division were added, as the team had to stay in Rotuma Island for at least a week until they returned to Suva due to the limited flights. All class 1–8 students of the selected schools in the Eastern Division whose parents consented via signing a consent form, were enrolled. This would satisfy WHO’s recommendation of 5–10 schools per ecological zone and a sample size of 50 students per school in all four Divisions [3].

**Table 1:** Number of selected schools and estimated sample sizes for the STH prevalence assessment in the 4 Divisions of Fiji, 2014–2015 [18].

		Western	Central	Northern*	Eastern
LF-TAS	Number of schools selected	77	82	50	-
STH prevalence assessment	Number of schools selected	30**	20**	20**	24
	Estimated sample size	1,692	1,479	724	1,902

*The Taveuni sub-Division is not included.

**Subsampled from LF-TAS schools

For the schools selected via LF-TAS in the three Divisions, questionnaires on demographic and WASH-related matters, in addition to the consent form, were sent to homes so that parents/guardians could fill in the questionnaire on behalf of the students. Since the team only stayed briefly in the islands of the Eastern Division, the questionnaires were not provided, as there was limited time to verify the information for the team members. All students were instructed to bring the signed consent forms and their stool containers half-filled with their fresh morning stool on the designated survey date; the stool samples were collected and kept in cool boxes until they were sent to the laboratory at the Fiji Center for Communicable Diseases Control (FCCDC), where samples were refrigerated until being prepared for Kato-Katz (KK) thick smears and the formol-ether-acetate concentration technique (FEC). The latter was used for the samples collected via LF-TAS only.

### Stool examination

Parasitologic diagnosis of STH was performed using KK smears prepared from a single stool sample (41.7 mg of stool per smear), described as a ‘cellophane fecal thick smear’ in the WHO laboratory guidelines [19]. The FEC, also known as ‘the concentration technique’ [19] was also applied to stool samples collected via LF-TAS; if stool samples were insufficient for both tests, then the FEC method was preferred to the KK smear. A positive STH infection is identified by the presence of helminth ova in the stool in either the KK smear or FEC test. Results of the KK examination were expressed as eggs per gram of feces (EPG), and infection intensities were categorized using the WHO guidelines [3]. Microscopic examination was conducted primarily by the parasitology laboratory at the FCCDC and supervised by technical experts, who re-examined 5% of slides independently for quality control purposes.

### Data management and statistical analysis

Demographic data of the children, and the results of the parasitologic examinations, were entered into a Microsoft Office Excel spreadsheet 2007 (Microsoft). Statistical analyses were conducted with the STATA Release 12 (College Station, TX: StataCorp LP). Samples were weighted according to the proportion of the sub-Divisional population in each Division as well as to the clustering effect at schools [20]. Point prevalence estimates and their confidence intervals were calculated using a logit transform to log 10, so that the endpoints lay between 0 and 1. In order to achieve 95% confidence intervals (CI) by category (locality, sex, or age groups) at the *P*<0.05 significance level [21], F ratios were obtained via adjusted Wald tests to explore whether point prevalence estimates were the same across different categories. The geometric mean of Williams was selected as the measure of central tendency for infection intensity values [22]. Comparisons and computations of 95% CIs of the geometric mean of EPG were conducted on the logs of the EPG values.

### Analysis of factors associated with STH

Data collected via a structured questionnaire in 3 Divisions via LF-TAS included: basic demographic information such as age and sex, individual behaviors associated with STH such as handwashing, shoe-wearing, and the use of utensils for meals, and household level water sources and the type of sanitary facilities. Parents/guardians were requested to evaluate frequencies of these behaviors based on their judgment by selecting one of three choices: not at all, not always but sometimes, or usually. For students’ primary water source and the type of sanitation facilities at home, options were provided following the Fijian Ministry of Health and Medical Services’ environmental standards to classify the relevant terms: (1) main source of water either to be spring, well, rainwater tanks, piped water into household from private or local source, or piped water directly from the Fiji Water Authority; and (2) the type of home latrine either to be river, bush, pit latrine, water-seal or pour-flush. Lastly, parents/guardians also were asked whether they could recall their children took a deworming or hookworm medication ever. For the school level water and sanitation characteristics, the school headmaster was interviewed to choose an option to report the type of their primary water source and the type of sanitation facilities at school.

For the analysis of the associations between the demographic and WASH characteristics and the odds of being STH-infected in the three Divisions surveyed via LF-TAS, a multi-level logistic regression model was applied accounting for intra-cluster correlation among the students attending the same school [23]. A random intercept logistic model was fitted for a generalized linear mixed model with random effects based on a logit link function for the estimates of the log odds of being STH-infected. Bivariate and multivariate analyses of associations between covariates and outcome variables, namely any STH, *Ascaris* spp. or hookworm infection, were undertaken, and factors associated with inter-school variation in infection prevalence were also explored. A manual stepwise forward logistic regression of significant variables was used to find the best predictive models, with P<0.1. We examined school-level effects on STH prevalence by adjusting for the effects of differences in the distribution of individual-level factors between schools. Using multi-level logistic regression models, school-level variance was evaluated for the different characteristics of the study participants.

### Ethics statement

The study was approved by the Fiji Ministry of Health and Medical Services National Health Research Committee, and the Ethical Review Board of Liverpool School of Tropical Medicine (14–01). Participation was fully voluntary, and parents were requested to sign a consent form, which was provided in three local languages, if they wished their children to take part in the study. Children were allowed to opt-out at any time during the survey.

## Results

### Demographic characteristics of the study participants

**Fig 2:**
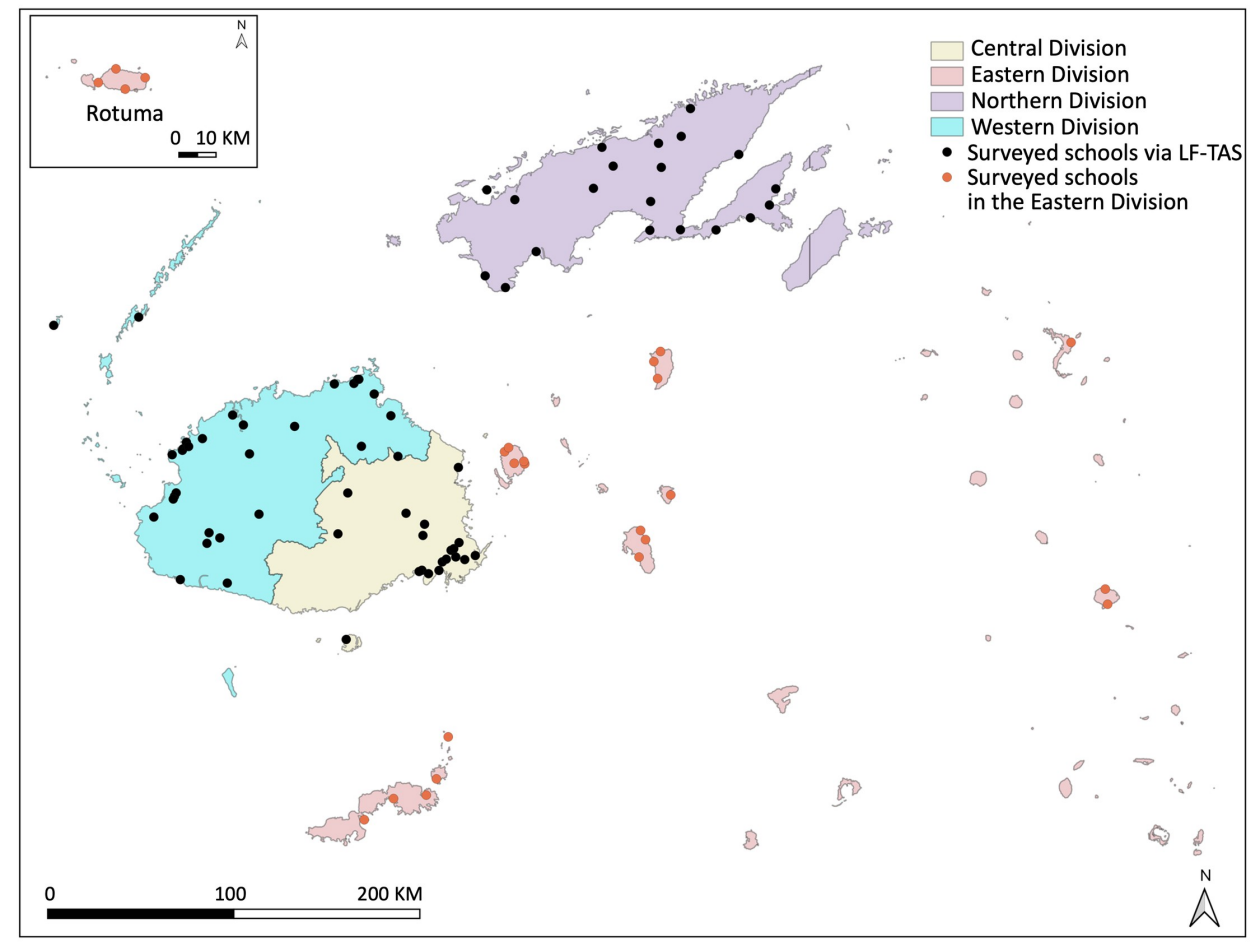
Sketch map of selected schools for the STH prevalence assessment in the four* Divisions of Fiji * The Taveuni sub-Division is not included. Data source; GADM database of Global Administrative Areas, August 2019. http://www.gadm.org/.

In total, 53.2% (1,890/3,551) of class 1 and 2 students in 69 out of 70 targeted schools, excluding one special school for the disabled, participated in the STH prevalence assessment via LF-TAS, where 1,839 stool samples were collected (Fig 2). In the Eastern Division, stool samples of 927 primary school students from class 1 to class 8 in 24 schools were collected out of 1,902, among them 248 samples were from class 1 and 2 students (Table 2). The demographic characteristics of the class 1 and 2 study participants in the 4 Divisions are shown in Table 2, and the demographic characteristics of the class 3–8 students in the Eastern Division are shown in Table 3.

**Table 2:** Demographic characteristics of class 1 and 2 students in the 4 Divisions of Fiji, 2014–2015.

Characteristic	Western Division	Central Division	Northern Division*	Eastern Division
	(n = 914)	(n = 526)	(n = 399)	(n = 248)
Response rate	74.5%	64.5%	61.6%	46.3%
Number and proportion** by current age (years) (95% CI)				
4–5	36 4.2% (2.6–6.6)	0 0.0%	2 0.8% (0.2–3.0)	3 0.6% (0.1–2.8)
6–7	829 90.6% (87.5–93.0)	413 89.3% (77.2–95.4)	383 95.4% (87.8–98.4)	216 82.8% (74.7–89.7)
8–10	37 5.3% (3.7–7.5)	37 10.7% (4.6–22.8)	14 3.8% (1.2–11.1)	60 16.7% (11.0–24.5)
Proportion of females** (95% CI)	46.8% (42.2–51.4)	48.5 (40.5–56.5)	50.8% (45.8–55.8)	37.0% (26.8–49.5)

*The Taveuni sub-Division is not included.

**All proportions are weighted based on the proportion of sub-Divisional per Divisional population sizes.

**Table 3:** Demographic characteristics of class 3 and 8 students in the Eastern Divisions of Fiji, 2015.

Characteristic	Class 3–4	Class 4–6	Class 7–8
	(n = 259)	(n = 207)	(n = 213)
Response rate	52.2%	46.3%	46.9%
Number and proportion* by current age (years) (95% CI)			
6–7	9 3.8% (1.2–11.5)	1 0.2% (0.0–2.0)	5 1.0% (0.2–5.0)
8–10	239 93.9% (88.2–96.9)	70 23.4% (15.2–34.1)	0
11–13	10 2.2% (0.7–6.5)	134 74.2% (64.8–81.8)	144 68.9% (49.9–83.1)
14–17	1 0.2% (0.0–1.4)	2 2.2% (0.4–11.6)	64 30.2% (16.0–49.5)
Proportion of females* (95% CI)	45.4% (35.2–56.0)	51.1% (34.0–68.0)	52.3% (46.8–57.8)

*All proportions are weighted based on the proportion of sub-Divisional per Divisional population sizes.

### Geographical distribution of STH in Fiji

At the national level, the any STH prevalence among class 1 and 2 students assessed via KK smear was 10.5% (95% CI: 6.9–15.5); most of the STH were *Ascaris* spp. (Table 4) and the prevalence of moderate-to- heavy intensity infection with *Ascaris* spp. was 3.7% (95% CI: 2.1–6.5).

**Table 4:** Prevalence* and intensity of STH by species among class 1 and 2 students in the 4 Divisions of Fiji, 2014–2015.

	Western Division	Central Division	Northern** Division	*P-*value***	Eastern Division****	All****
	(n = 914)	(n = 526)	(n = 399)		(n = 248)	(n = 2,087)
Any STH						
Prevalence (%) (95% CI)	5.9 (3.7–9.3)	20.7 (8.7–41.4)	16.8 (10.4–25.8)	**0.0172**	13.3 (6.4–25.6)	10.5 (6.9–15.5)
Range of sub-Divisional level prevalence (%)	0.0–11.1	3.8–38.2	9.0–19.1	-	0.0–27.0	0.0–38.2
Mono STH						
*Ascaris*						
Prevalence (%) (95% CI)	4.3 (2.8–6.7)	18.2 (6.7–40.6)	5.1 (3.3–7.9)	**0.0061**	13.3 (6.4–25.6)	9.8 (6.3–14.8)
Low intensity infection (%) (95% CI)	2.5 (1.5–4.3)	9.6 (3.7–23.0)	3.1 (1.6–6.0)	**0.0014**	7.7 (3.7–15.4)	6.1 (3.9–9.3)
Moderate/heavy intensity infection (%) (95% CI)	1.3 (0.6–2.7)	4.2 (0.9–17.1)	1.7 (0.7–3.9)	0.1787	5.5 (2.3–12.7)	3.7 (2.1–6.5)
Geometric mean eggs per gram (95% CI)	1382.2	3528.3	832.3	0.41	2480.7	2341.0
	(611.1–3126.3)	(1929.6–6451.6)	(302.2–2292.1)		(1433.4–4293.3)	(1588.2–3450.7)
Range of sub-Divisional level prevalence (%)	0.0–8.0	0.0–35.5	0.0–6.5	-	0.0–27.0	0.0–35.5
Hookworm						
Prevalence (%) (95% CI)	2.1 (0.7–6.1)	2.5 (1.1–5.8)	12.8 (7.3–21.7)	**0.0004**	0.7 (0.2–2.5)	0.8 (0.2–2.5)
Low intensity infection (%) (95% CI)	1.3 (0.4–4.9)	1.1 (0.3–3.7)	1.9 (0.8–4.5)	0.806	0.7 (0.2–2.5)	0.8 (0.2–2.5)
Moderate/heavy intensity infection (%) (95% CI)	0	0	0	-	0	
Geometric mean eggs per gram (95% CI)	145.5 (19.1–1109.2)	-	24	-	40.1 (9.15–176.2)	72.5 (44.4–118.2)
Range of sub-Divisional level prevalence (%)	0.0–6.0	0.0–9.5	9.0–13.9		0.0–2.1	0.0–13.9
*Trichuris*						
Prevalence (%) (95% CI)	0	0	0		0	
Dual STH						
*Ascaris* and hookworm						
Prevalence (%) (95% CI)	0.7 (0.2–2.1)	0	1.2 (0.6–2.5)	0.1666	0.7 (0.2–2.5)	0.5 (0.2–1.2)

*All point estimates and CIs are weighted based on the proportion of sub-Divisional per Divisional population sizes.

**The Taveuni sub-Division is not included.

***Across the Western, Central, and Northern Divisions, and in bold fonts when < 0.05

**** Based on the Kato-Katz thick smear technique only.

#### STH prevalence assessment by LF-TAS

By coproscopy, overall 12.1% (95% CI: 7.3–19.4) of children were infected in the Western, Central, and Northern Divisions, either with *Ascaris* (8.7%, 95% CI: 4.3–16.6) or hookworm (3.9%, 95% CI: 2.3–6.6), and no *Trichuris* infections were found by KK smear and/or FEC. For the cases where egg counts were available via KK, low- intensity *Ascaris* infections were more prevalent (4.8%, 95% CI: 2.4–9.2) than moderate or high-intensity infections (2.2%, 95% CI: 0.9–5.5). For hookworm, there were no moderate or high-intensity infections, and only low-intensity infections (1.3%, 95% CI: 0.6–3.0) (Table 4).

**Fig 3:**
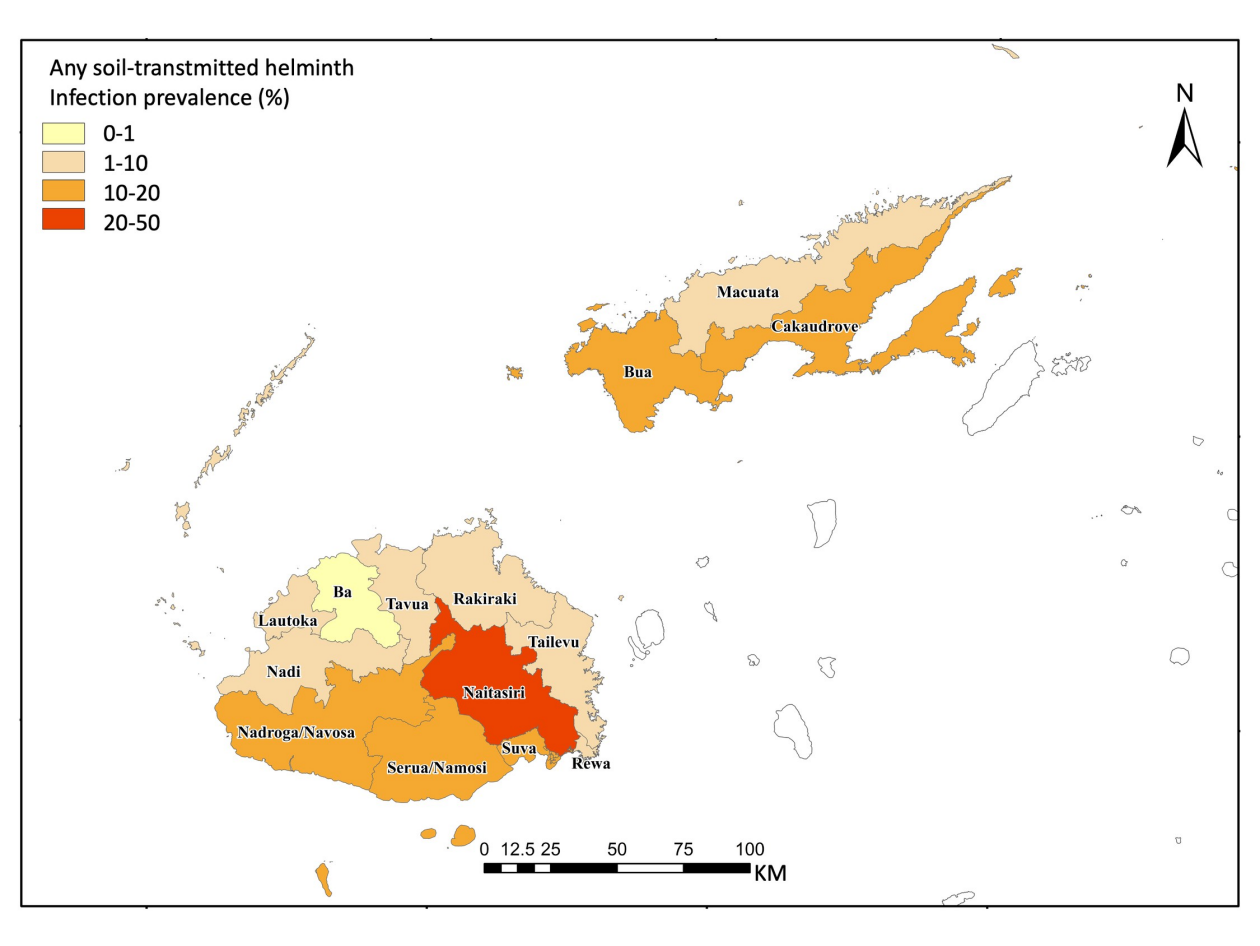
Sketch map of sub-Divisional “any STH” prevalence levels in the Western, Central, and Northern* Divisions, 2014–2015 *Except the Taveuni sub-Division.

**Fig 4:**
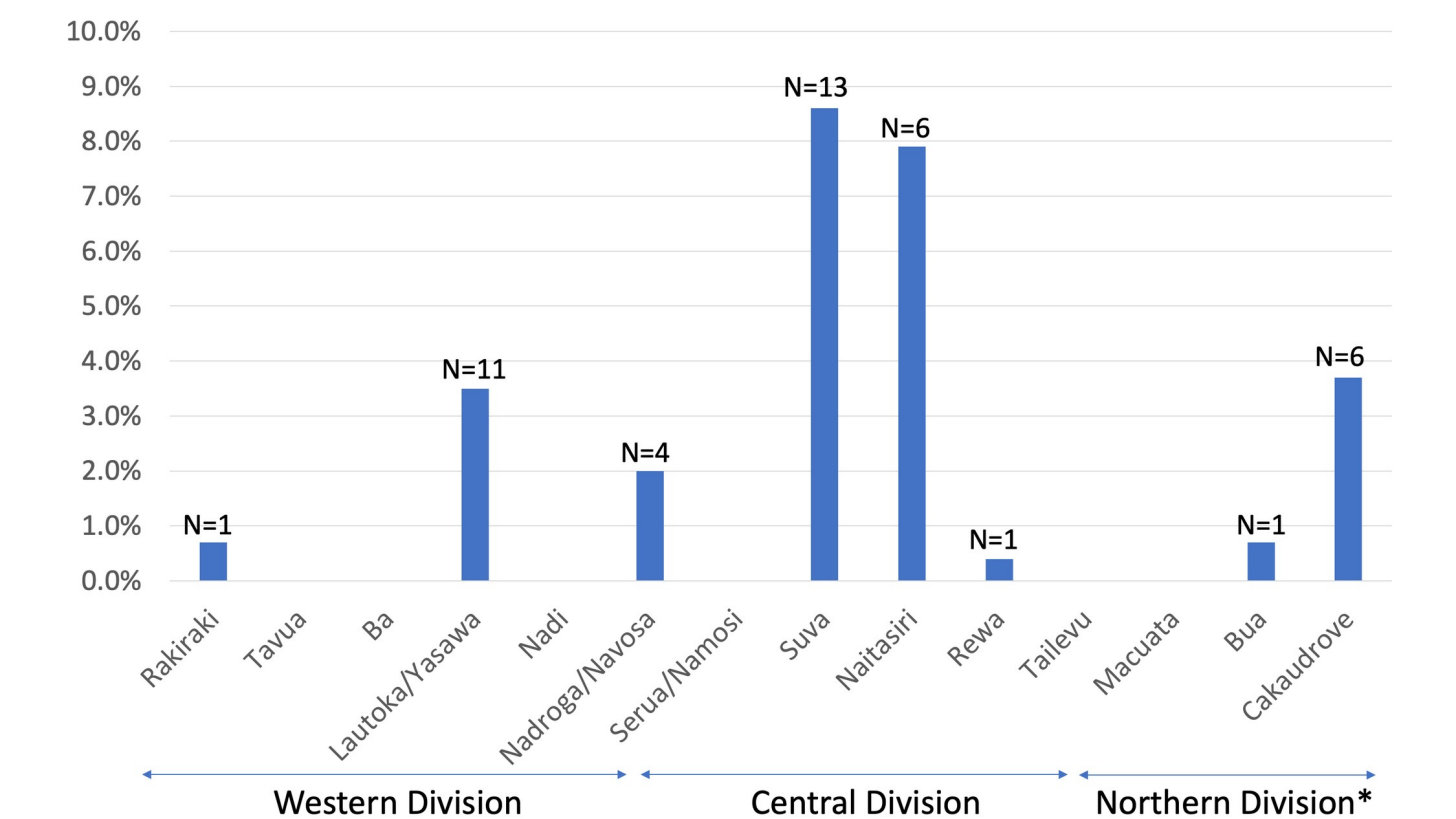
Distribution of sub-Divisional prevalence levels of “any moderate or heavy intensity STH” in the Western, Central, and Northern* Divisions, 2014–2015 *Except the Taveuni sub-Division.

Prevalence levels for any STH differed across sub-Divisions (*P* = 0.0014), with the highest frequency, up to 38.2% (95% CI: 17.8–63.8), in the Nataisiri sub-Division of the Central Division (Fig 3). The Suva sub-Division of the Central Division had the highest point prevalence, up to 8.6% (95% CI: 2.5–25.6), of any moderate or heavy intensity STH, but estimated prevalence levels did not differ across sub-Divisions (P = 0.8759) (Fig 4). In more than half (9/14) of the sub-Divisions the point estimates for any moderate or heavy-intensity STH were below 1%, which is the elimination goal for STH as a public health problem (WHO 2012a) (Fig 4).

#### STH prevalence assessment in the Eastern Division

The overall estimated prevalence of any STH of class 1 and 2 students was 13.3% (95% CI: 6.4–25.6). Every identified hookworm infection was also positive for *Ascaris*, and no *Trichuris* infections were discovered (Table 5). Prevalence levels of any STH and *Ascaris* infections were highest in class 1–2 students and lowest in class 7–8 students (*P* = 0.0216), showing that prevalence levels were not the same across class groups (Table 5). In terms of infection intensity, both low-intensity and moderate to heavy-intensity *Ascaris* infections were more frequent in class 1–2 than in the other groups, but without statistical significance (Table 5). The prevalence of hookworm infections did not differ across class groups and only one case of *Trichuris* spp. infection was found (in a class 5–6 student).

**Table 5:** Prevalence* and intensity of STH by species among class 1–8 students of the Eastern Divisions of Fiji, 2015.

	Class groups				*P-*value**
	Class 1–2	Class 3–4	Class 5–6	Class 7–8	
	(n = 248)	(n = 259)	(n = 207)	(n = 213)	
*Ascaris* infection					
Prevalence (%) (95% CI)	13.3	7.3	9.4	5.6	**0.0216**
	(6.4–25.6)	(2.8–17.3)	(3.1–24.6)	(1.9–15.9)	
Low intensity infection (%) (95% CI)	7.7	4.6	5.8	3.5	0.1356
	(3.7–15.4)	(1.9–10.9)	(2.1–15.1)	(1.1–10.5)	
Moderate/heavy intensity infection (%) (95% CI)	5.5	2.3	3.5	2.2	0.0656
	(2.3–12.7)	(0.5–10.2)	(1.0–12.0)	(0.4–11.2)	
Geometric mean epg	2480.7	1665.4	1967.6	2708.7	0.995
	(1433.4–4293.3)	(548.8–5053.5)	(546.8–7079.5)	(913.6–8031.2)	
Hookworm infection				
Prevalence (%) (95% CI)	0.7	0.2	0.8	0.2	0.3276
	(0.2–2.5)	(0.0.-2.0)	(0.2–2.9)	(0.0–1.8)	
Low intensity infection (%) (95% CI)	0.7	0.2	0.8	0.2	0.3276
	(0.2–2.5)	(0.0.-2.0)	(0.2–2.9)	(0.0–1.8)	
Geometric mean epg	40.1	72	83.4	48	0.385
	(9.15–176.2)		(63.5–109.5)		
*Trichuris* infection				
Prevalence (%) (95% CI)	-	-	0.2	-	-
			(0.0–1.9)		
Low intensity infection (%) (95% CI)	-	-	0.2	-	-
			(0.0–1.9)		
Geometric mean epg	-	-	24	-	-

*All point estimates and CIs are weighted based on the proportion of sub-Divisional per Divisional population sizes.

**Across 4 class groups, and in bold fonts when < 0.05

### Factors associated with STH in the Western, Central, and Northern Divisions of Fiji

#### Individual, household, and school-level WASH characteristics of the study participants

More than half of students were reported to wash their hands before eating or after toilet use, to use utensils during meals, and to wear shoes, as denoted by ‘usually’ in S2 Table. In one-third of the study participants, the parents/guardians answered that they could recall previous ingestions of deworming medication by their children. The majority of households had either water-seal or pour-flush latrines and use of pit latrines or open spaces such as a river or bush were overall infrequent. In schools, the most frequently reported main source of water was water piped from the Fiji Water Authority, while in the Northern Divisions it was water piped from private or local sources. That was also the only Division which reported a school without on-site toilets and open space toilet use by students.

#### Association between children’s demographic and WASH characteristics and STH

After exploring the best model to describe the dataset obtained, a two-level model (students at level 1 nested within a school at level 2) was fitted. Current age and class were closely related (*ρ* = 0.81) and only current age was retained in the later stages of the analysis. Using multilevel logistic regression models, school-level variances for the different characteristics of children were evaluated. In summary, a comparison between the null model and the random intercept model indicated that 66% of the school-level variation was accounted for by the individual characteristics considered in the model.

When school level variation was allowed in the multivariate model, 4-6-year-old children were associated with higher odds of any STH than 10-year-olds (Table 6). Children who were reported to wash their hands and to wear shoes ‘usually’ had lower odds of being “any STH eggs” positive than students who were reported to wash their hands and to wear shoes less frequently such as ‘not always but sometimes’ or ‘not at all’. As for the main water source at home, those having piped water from the Fiji Water Authority had lower odds for any STH than children whose home water sources were either piped water from private or local sources or other. These explanatory variables with statistically significant ß coefficients were then allowed to vary, one by one, across schools, but no variable had significantly varying effects on any STH prevalence across schools, confirming the final model to be a random-intercept model rather than a random coefficient model. Species-specific odds ratios are also presented in Table 6.

**Table 6:** Associations between students’ demographic and WASH characteristics and STH prevalence levels by species.

Characteristic	Any STH*				*Ascaris* infection*				Hookworm infection*			
	Crude odds ratio	*P*-value	Adjusted** odds ratio	*P*-value	Crude odds ratio	*P*-value	Adjusted** odds ratio	*P*-value	Crude odds ratio	*P*-value	Adjusted** odds ratio	*P*-value
	(95% CI)		(95% CI)		(95% CI)		(95% CI)		(95% CI)		(95% CI)	
By current age (years)												
4~6	1.7	**0.005**	1.8	**0.008**	1.98	**0.004**	2.18	**0.007**	1.37	0.249	** **	** **
	(1.18-2.46)		(1.17-2.78)		(1.24-3.17)		(1.24-3.81)		(0.80-2.33)		** **	
7~10	reference		reference		reference		reference		reference			
By sex												
Female	0.88	0.452			1.06	0.782			0.65	0.097		
	(0.63-1.23)				(0.70-1.60)				(0.39-1.08)			
Male	reference				reference				reference			
By handwashing behavior before eating or after toilet use												
Yes, usually	0.57	**0.02**	0.68	0.139	0.54	**0.039**	0.6	0.108	0.73	0.35		
	(0.36-0.92)		(0.41-1.13)		(0.30-0.97)		(0.32-1.12)		(0.38-1.41)			
Not always but sometimes	reference		reference		reference		reference		reference			
Not at all												
By utensil use during meals												
Yes, usually	0.91	0.67			0.84	0.536			1.17	0.642		
	(0.59-1.41)				(0.49-1.45)				(0.62-2.19)			
Not always but sometimes	reference				reference				reference			
Not at all												
By shoe-wearing behavior												
Yes, usually	0.45	**0.001**	0.54	**0.022**	0.49	**0.023**	0.57	0.097	0.5	**0.033**	0.6	0.133
	(0.28-0.72)		(0.32-0.91)		(0.26-0.90)		(0.29-1.11)		(0.27-0.95)		(0.31-1.17)	
Not always but sometimes	reference		reference		reference		reference		reference		reference	
Not at all												
By recalling deworming medication ingestion by the child												
Yes	0.9	0.652			1.54	0.138			0.47	**0.038**	0.51	0.085
	(0.57-1.43)				(0.87-2.71)				(0.23-0.96)		(0.24-1.10)	
No or no answer	reference				reference				reference		reference	
By main water source at home												
Piped water, Fiji Water Authority	0.47	**0.019**	0.48	**0.026**	0.6	0.209			0.35	**0.019**	0.42	**0.049**
	(0.25-0.88)		(0.25-0.92)		(0.28-1.33)				(0.15-0.84)		(0.17-1.00)	
Piped water, private or local	reference		reference		reference				reference		reference	
Others (Rainwater tank, borehole, river or stream)												
By home latrine type												
Water-seal/pour-flush	0.59	0.068			0.76	0.511			0.47	**0.038**	0.57	0.138
	(0.33-1.04)				(0.34-1.70)				(0.23-0.96)		(0.27-1.20)	
Pit latrine	reference				reference				reference		reference	
River or bush												

*Outcome variables among children participating in the STH prevalence assessment through LF-TAS were defined as follows: (1) Any STH indicates being positive for ova of at least one species, marked as a binary variable (zero or one); and (2) *Ascaris* infection and hookworm infection were denoted as positive when ova of *Ascaris* or hookworm were found, and marked as a binary variable (zero or one).

**Adjusted for all other variables.

*** *P*-values in bold fonts when < 0.05

## Discussion

The usefulness of LF-TAS in assessing the epidemiological profile of STH as a platform for determining PCT strategies upon stopping LF-MDA has been clearly demonstrated in other countries [24–26]. This experience led to the recommendation in the WHO guidelines to assess the epidemiology of STH during LF-TAS [2]: the target population for the STH survey in conjunction with LF-TAS is designated as 8-10-year-olds for school-based surveys and 6-7-year-olds for household surveys. Nevertheless, our study is unique in the sense that the STH prevalence assessment took place at exactly the same time as the school-based LF-TAS, with the same class 1–2 students as target individuals. This avoided the burden of mobilizing and running several different teams at the same time and proved that adding the STH component is feasible and efficient in settings with limited resources.

Our survey of 69 primary schools across the Western, Central, and Northern Divisions using LF-TAS and of 24 schools in the Eastern Division showed that the national STH prevalence in school-aged children of Fiji was now close to 10%. The residual infections were mainly composed of *Ascaris* spp., followed by hookworm, while *Trichuris* infections were rare. Our recent appraisal contrasts with an earlier survey conducted in 2005, where only 258 school children from five villages in Taveuni Island were examined [27] and the overall prevalence of *Ascaris*, hookworm, and *Trichuris* infections were 33%, 14%, and 17%, respectively. Though children in the same localities were not included in our assessment, it is likely that the large-scale PCT, especially the community-based LF-MDA, has reduced STH in the two major islands of Fiji [25].

The most notable finding from our study, however, is the large variation in STH prevalence at sub-Division-level. This is consistent with recent findings from a similar study in Kenya [28], where the impact of PCT was assessed and varied markedly by county. Whilst some uncertainties remain, as neither pre-LF-MDA baseline data nor school-level treatment coverages are available, it is likely that variable performance of community-based LF-MDA by locality influenced the epidemiologic situation for STH, and that there were also innate geographical differences in the underlying intensity of transmission that influenced local reinfection rates between the treatments provided by community-based LF-MDA [28].

Though STH is still widely prevalent, epidemiologic investigations in Oceania including Fiji are limited, and we have here presented an in-depth investigation of the factors associated with STH in Fijian school children living on the two main islands. It is well known that the risk of infection with *A*. *lumbricoides* and *T*. *trichiura* decreases with age among SAC, while it increases with age for hookworm [29,30]. In our study population we observed that increasing age reduced the risk of any STH, largely because *Ascaris* infections were dominant. This is also in line with the findings of our study in the Eastern Division, where a wider age range of children, from classes 1 to 8, was included. One plausible explanation is that older children adopt more-hygienic behaviors than their younger counterparts, but we did not measure this association among the wider population age groups in our study. Another explanation might be that the older students had been exposed more frequently to albendazole via community-based LF-MDA and school-based deworming over the previous years.

A statistically significant difference existed between children regularly wearing shoes and children who did not wear shoes or did so only occasionally. The former had only half the likelihood of being infected by any STH, implying that shoe-wearing is associated with lower prevalence levels of any STH. A recent meta-analysis provided evidence of a strong association between wearing shoes and lower odds of hookworm infection [16,31], as well as of any STH [16], as seen in our study population. The proportion of children who did not wear shoes regularly maybe have been higher in reality, considering that this is shoe-wearing reported by caregivers rather than based on direct observation. These findings underscore the need for studies to explore whether local populations are unaware of the health benefits of wearing shoes or whether there are socio-economic barriers, e.g. poverty, that lead children to wear shoes occasionally or not at all in this Fijian setting [32].

Another major finding in our study is that having the main water supply from the Fiji Water Authority was associated with lower odds of any STH and hookworm infection compared with having it from any other source. This is similar to what was reported in a recent meta-analysis, where using treated water (filtered or boiled) was associated with a lower likelihood of having any STH infection [16]. However, there were not sufficient studies to be able to conduct a similar meta-analysis for hookworm infection [16]. Water is a critical component of WASH resources [33], and according to our observations in the field, having the main water supply from the Fiji Water Authority, which is piped and treated water, provides not only quality-controlled water but also fewer interruptions of supply than being supplied from other sources. Therefore, being supplied by the Fiji Water Authority may be a surrogate for other WASH characteristics, such as availability of functioning washing stations, rather than being causally associated with infection [28].

As the current study suggests, Fiji is progressing well towards the goal of eliminating STH as a public health problem, as overall moderate and heavy intensity any STH is just around 1% in many of sub-Divisions of the two main islands [34], even though data obtained prior to the commencement of PCT via community-based LF-MDA are insufficient to be able to conclude whether this low level of prevalence represents a direct impact of the PCT, or whether it was low before the interventions. In the new global guidelines for controlling STH by PCT, the use of annual or biannual single-dose albendazole (400 mg) or mebendazole (500 mg) is recommended as a public health intervention for all young children (12–23 months of age) as well as pre-SAC (24–59 months of age) and SAC living in areas where the baseline prevalence of any STH is 20% or higher among children [12]. Hence the country would not require a large-scale PCT program, as only low-intensity infection and low morbidity are expected where the prevalence of any STH is below 20% at the national level. Nevertheless, given the varying levels of prevalence of any STH at the sub-National level, the control program can now consider applying PCT in any Division or sub-Division where the prevalence level >20%. As an example, the Central Division, or the Nataisiri sub-Division of the Central Division itself, would need to implement annual deworming using single dose albendazole as a public health intervention for all young children, pre-SAC as well as SAC [12]. The treatment option of having a Division or a sub-Division as implementation unit is only possible because we have provided up-to-date epidemiologic information of higher resolution that the national programme can refer to. In addressing all at-risk population groups as proposed in the new guideline, it is also recommended to explore the STH status of non-pregnant adolescent girls and non-pregnant women of reproductive age. This is critical for deciding whether or not to implement deworming as a public health intervention for these groups, considering that annual or biannual anthelminthic treatment is now recommended for this population [12].

The program should also further explore the best delivery strategy for achieving the global goal of 75% PCT coverage for all young children in the identified implementation unit, given the recent progress in the country’s NTD control and elimination programs, especially for LF. First, like Burkina Faso, Fiji is a good candidate for pursuing the STH control goal via community-based LF-MDA [25,35] in areas with remaining LF endemicity. Second, school-based mass deworming could be prioritized, either using a stand-alone ad-hoc approach or in combination with the perennial students’ health check-ups. Finally, implementation research may be needed to explore the best delivery channels, especially for young children (12–23 months of age and pre-SAC); for instance, should it be facility- or community-based? That is a critical decision in a post-LF-MDA surveillance setting like Fiji, where community-based LF-MDA rounds, especially via house-to-house drug distribution, have been stopped in the most part.

Until recently, the emphasis has been on control of STH, rather than on interrupting transmission. Moreover, PCT recommendations are not directly linked to the elimination target but to the overall STH prevalence levels of the implementation unit, mostly defined at the national level. Historical data show that interruption of STH transmission is possible even at the national level [36], in such cases as Japan [37], Republic of Korea [38], and Taiwan [39]. Critical components identified were mass deworming or selective chemotherapy of infected schoolchildren, political support by legislative measures, and strong inter-sectoral collaboration coupled with parallel improvements in socioeconomic status and access to water and sanitation [36]. It is now likely that elimination of STH as a public health problem in Fiji would be feasible by following a similar approach, given that our study has shown that the intensity of transmission is low in the two main islands, and suitable delivery platforms, especially for SAC, are available as in-country resources. Supportive household environments and strong health systems should therefore back up an inter-sectoral approach to achieve local elimination of STH as a public health problem [36].

There are several limitations to this study. First, the age group selected for the STH prevalence assessment through LF-TAS was limited to class 1 and 2 schoolchildren, and the epidemiological profile of infection in older children, or those who do not attend school, may be different. Since the risk of hookworm infection may be higher in the adult population [35], we may have underestimated the true burden of the diseases. We tried to overcome this limitation by expanding the age groups in the Eastern Divison and revealing the association between age and *Ascaris* infection prevalence. However; it was not feasible to do the same for hookworm infections, as the number of positive cases was too low. Also, the KK technique was not applied on site for most of the stool samples not collected in Viti Levu, and this may have impacted detection of hookworm eggs in those samples [40]. All in all, the results may represent the situation for *Ascaris* and *Trichuris* infections but may not represent that for hookworm in all the populations at risk [25]. Also, it may be necessary to assess STH status among pre-SAC and adults in the communities, to explore whether other factors are involved. As for the analysis of factors associated with STH, this was restricted to the variables that we had measured and may not capture the whole picture of local STH transmission dynamics [41]. Since ours was the first attempt to use LF-TAS as a survey platform to assess STH epidemiology in Fiji, we used a simple questionnaire for collecting WASH-related data, to ensure that surveys were practicable and efficient. In this regard, the measurements of WASH characteristics were based on care-givers’ reports, rather than direct observation by the survey team; therefore, it is possible that the frequencies of desirable hygiene practices were over / under-estimated, and that the functioning and accessibility of water and sanitation infrastructures were not appropriately reflected in the answers. It may be useful to directly assess additional detailed WASH variables in the near future, if there are opportunities for other public health programs to carry out further surveys. Lastly, measurement of the WASH covariates was undertaken following infections, whereas causality is time-bound, and causes should precede dependent effects. Thus, the associations reported here may not be causal [42]. We also assumed that the value of a covariate measured at a given time point had been consistent over time, which may not always be realistic, especially for individual hygiene practices.

### Conclusions

By adding stool sample collection to LF-TAS in the Western, Central, and Northern Divisions and organizing an independent STH prevalence assessment in the Eastern Division, we have been able to shed new light on the up-to-date epidemiologic profile of STH in a country largely at the stage of post-LF-MDA surveillance. This study provides important information for the national STH control program of Fiji regarding essential requirements for delivering effective control strategies over the next 4–6 years, and where to do so. Following the new WHO global guidelines, Fiji will now be required to implement PCT either at Divisional or sub-Divisional level, rather than at the national. It seems to be feasible for Fiji to achieve the global goal of eliminating STH as a public health problem if multi-sectoral approaches are sustained and complemented with improvements in water, sanitation, and hygiene conditions. The results reported here were subsequently acknowledged by the national health authorities in setting up the baseline values of the core program indicators for monitoring whether the program is on track in terms of achieving the integrated NTDs control targets, in line with the relevant SDG goals [43]. LF-TAS could be used as a monitoring and evaluation tool for other NTD programmes.

## Supporting information

S1 Table Checklist. STROBE checklist.
(DOCX)

S2 Table Distribution of individual, household, and school WASH characteristics of the study participants and STH prevalence in the Western, Central and Northern Divisions of Fiji, 2014–2015. (DOCX)

## References

[1] WHO. WHO | Soil-transmitted helminth infections [Internet]. 2017 [cited 13 Dec 2017]. Available: http://www.who.int/mediacentre/factsheets/fs366/en/

[2] WHO. Assessing the epidemiology of soil-transmitted helminths during a transmission assessment survey (TAS). 2015.

[3] WHO. Helminth control in school-age children. 2nd ed Geneva: World Health Organization; 2011.

[4] WPRO. WPRO | Lymphatic filariasis [Internet]. 2015 [cited 7 Aug 2019]. Available: http://www.wpro.who.int/topics/lymphatic_filariasis/en/

[5] Kim SH, Rinamalo M, Rainima-Qaniuci M, Talemaitoga N, Kama M, Rafai E, et al Island-Wide Surveillance of Gastrointestinal Protozoan Infection on Fiji by Expanding Lymphatic Filariasis Transmission Assessment Surveys as an Access Platform. Am J Trop Med Hyg. 2018;98: 1179–1185. 10.4269/ajtmh.17-0559 29405101 PMC5928820

[6] Rinamalo M, Tuibeqa S, Rafai E, Kim SH. Mid-term Assessment Towards Elimination of Lymphatic Filariasis in Fiji, 2013. Fiji Journal of Public Health. 2014;2.

[7] WHO. WHO | PCT databank [Internet]. 2017 [cited 4 Aug 2019]. Available: http://www.who.int/neglected_diseases/preventive_chemotherapy/lf/en/

[8] Vasu K. Nationa Iron and Micronutrient Supplementation Project Annual Report 2014. 2015.

[9] Fletcher S, Caprarelli G, Merif J, Andresen D, Hal SV, Stark D, et al Epidemiology and geographical distribution of enteric protozoan infections in Sydney, Australia. J Public Health Res. 2014;3: 298 10.4081/jphr.2014.298 25343139 PMC4207027

[10] Bethani E, Goneyali S, Volavola I. Prevalence of intestinal helminth infection in Fiji. Pac Health Dialog. 1998;5: 74–75.

[11] Hughes RG, Sharp DS, Hughes MC, Akau’ola S, Heinsbroek P, Velayudhan R, et al Environmental influences on helminthiasis and nutritional status among Pacific schoolchildren. Int J Environ Health Res. 2004;14: 163–177. 10.1080/0960312042000218589 15203448

[12] WHO. WHO | Preventive chemotherapy to control soil-transmitted helminth infections in at-risk population groups [Internet]. 2017 [cited 29 Dec 2017]. Available: http://www.who.int/intestinal_worms/resources/9789241550116/en/29578660

[13] WHO. Accelerating work to overcome the global impact of neglected tropical diseases: a roadmap for implementation. 2012.

[14] Gazzinelli A, Correa-Oliveira R, Yang G-J, Boatin BA, Kloos H. A research agenda for helminth diseases of humans: social ecology, environmental determinants, and health systems. PLoS Negl Trop Dis. 2012;6: e1603 10.1371/journal.pntd.0001603 22545168 PMC3335881

[15] Strunz EC, Addiss DG, Stocks ME, Ogden S, Utzinger J, Freeman MC. Water, sanitation, hygiene, and soil-transmitted helminth infection: a systematic review and meta-analysis. PLoS Med. 2014;11: e1001620 10.1371/journal.pmed.1001620 24667810 PMC3965411

[16] FAO. Fiji country pasture/forage resource profiles [Internet]. 2009 [cited 11 Mar 2016]. Available: http://www.fao.org/ag/agp/agpc/doc/Counprof/southpacific/fiji.htm

[17] Fiji Bureau of Statistics. 2007 Census of Population [Internet]. 2009 [cited 17 Nov 2015]. Available: http://www.statsfiji.gov.fj/index.php/2007-census-of-population

[18] Fiji Ministry of Education, Fiji Education Management Information System (FEMIS) School enrollment data, 2015.

[19] WHO, editor. Basic laboratory methods in medical parasitology. Geneva: WHO; 1991.

[20] Levy PS, Lemeshow S. Sampling of populations: Methods and applications. 4th ed Hoboken, N.J: Wiley; 2008.

[21] Richardson JT. The analysis of 2 x 1 and 2 x 2 contingency tables: an historical review. Stat Methods Med Res. 1994;3: 107–133. 10.1177/096228029400300202 7952428

[22] Alexander N. Review: analysis of parasite and other skewed counts. Trop Med Int Health. 2012;17: 684–693. 10.1111/j.1365-3156.2012.02987.x 22943299 PMC3468795

[23] Kawachi I, Berkman LF, editors. Neighborhoods and Health. Oxford University Press; 2003 10.1093/acprof:oso/9780195138382.001.0001

[24] Chu BK, Gass K, Batcho W, ‘Ake M, Dorkenoo AM, Adjinacou E, et al Pilot assessment of soil-transmitted helminthiasis in the context of transmission assessment surveys for lymphatic filariasis in Benin and Tonga. PLoS Negl Trop Dis. 2014;8: e2708 10.1371/journal.pntd.0002708 24551267 PMC3923741

[25] Drabo F, Ouedraogo H, Bougma R, Bougouma C, Bamba I, Zongo D, et al Successful Control of Soil-Transmitted Helminthiasis in School Age Children in Burkina Faso and an Example of Community-Based Assessment via Lymphatic Filariasis Transmission Assessment Survey. PLoS Negl Trop Dis. 2016;10: e0004707 10.1371/journal.pntd.0004707 27163294 PMC4862685

[26] Knipes AK, Lemoine JF, Monestime F, Fayette CR, Direny AN, Desir L, et al Partnering for impact: Integrated transmission assessment surveys for lymphatic filariasis, soil transmitted helminths and malaria in Haiti. PLoS Negl Trop Dis. 2017;11: e0005387 10.1371/journal.pntd.0005387 28207792 PMC5332101

[27] Thomas M, Woodfield G, Moses C, Amos G. Soil-transmitted helminth infection, skin infection, anaemia, and growth retardation in schoolchildren of Taveuni Island, Fiji. N Z Med J. 2005;118: U1492 15937527

[28] Nikolay B, Mwandawiro CS, Kihara JH, Okoyo C, Cano J, Mwanje MT, et al Understanding Heterogeneity in the Impact of National Neglected Tropical Disease Control Programmes: Evidence from School-Based Deworming in Kenya. PLoS Negl Trop Dis. 2015;9: e0004108 10.1371/journal.pntd.0004108 26421808 PMC4589351

[29] Muller R. Worms and human disease. 2nd ed Wallingford, Oxon, UK: CABI; 2002.

[30] Gabrie JA, Rueda MM, Canales M, Gyorkos TW, Sanchez AL. School hygiene and deworming are key protective factors for reduced transmission of soil-transmitted helminths among schoolchildren in Honduras. Parasit Vectors. 2014;7: 354 10.1186/1756-3305-7-354 25091035 PMC4132920

[31] Ziegelbauer K, Speich B, Mäusezahl D, Bos R, Keiser J, Utzinger J. Effect of sanitation on soil-transmitted helminth infection: systematic review and meta-analysis. PLoS Med. 2012;9: e1001162 10.1371/journal.pmed.1001162 22291577 PMC3265535

[32] Paige SB, Friant S, Clech L, Malavé C, Kemigabo C, Obeti R, et al Combining Footwear with Public Health Iconography to Prevent Soil-Transmitted Helminth Infections. Am J Trop Med Hyg. 2017;96: 205–213. 10.4269/ajtmh.15-0910 27821692 PMC5239695

[33] Mogaji HO, Dedeke GA, Jaiyeola OA, Adeniran AA, Olabinke DB, Oluwole AS, et al A preliminary survey of school-based water, sanitation, hygiene (WASH) resources and soil-transmitted helminthiasis in eight public schools in Odeda LGA, Ogun State, Nigeria. Parasitology Open. 2017;3 10.1017/pao.2017.18

[34] WHO. Eliminating Soil-transmitted helminthiases as a public health problem in children. 2012.

[35] Anderson RM, Turner HC, Truscott JE, Hollingsworth TD, Brooker SJ. Should the Goal for the Treatment of Soil Transmitted Helminth (STH) Infections Be Changed from Morbidity Control in Children to Community-Wide Transmission Elimination? PLoS Negl Trop Dis. 2015;9: e0003897 10.1371/journal.pntd.0003897 26291538 PMC4546270

[36] Brooker SJ, Nikolay B, Balabanova D, Pullan RL. Global feasibility assessment of interrupting the transmission of soil-transmitted helminths: a statistical modelling study. Lancet Infect Dis. 2015;15: 941–950. 10.1016/S1473-3099(15)70042-3 25886799

[37] Kobayashi A, Hara T, Kajima J. Historical aspects for the control of soil-transmitted helminthiases. Parasitol Int. 2006;55 Suppl: S289–91. 10.1016/j.parint.2005.11.042 16376139

[38] Hong S-T, Chai J-Y, Choi M-H, Huh S, Rim H-J, Lee S-H. A successful experience of soil-transmitted helminth control in the Republic of Korea. Korean J Parasitol. 2006;44: 177–185. 10.3347/kjp.2006.44.3.177 16969055 PMC2532657

[39] WHO, editor. Preventive chemotherapy in human helminthiasis. 2006.

[40] Tarafder MR, Carabin H, Joseph L, Balolong E, Olveda R, McGarvey ST. Estimating the sensitivity and specificity of Kato-Katz stool examination technique for detection of hookworms, *Ascaris lumbricoides* and *Trichuris trichiura* infections in humans in the absence of a “gold standard”. Int J Parasitol. 2010;40: 399–404. 10.1016/j.ijpara.2009.09.003 19772859 PMC2829363

[41] Greenland K, Dixon R, Khan SA, Gunawardena K, Kihara JH, Smith JL, et al The epidemiology of soil-transmitted helminths in Bihar State, India. PLoS Negl Trop Dis. 2015;9: e0003790 10.1371/journal.pntd.0003790 25993697 PMC4439147

[42] Benjamin-Chung J, Nazneen A, Halder AK, Haque R, Siddique A, Uddin MS, et al The Interaction of Deworming, Improved Sanitation, and Household Flooring with Soil-Transmitted Helminth Infection in Rural Bangladesh. PLoS Negl Trop Dis. 2015;9: e0004256 10.1371/journal.pntd.0004256 26624994 PMC4666415

[43] WHO. WHO | Water sanitation and hygiene for accelerating and sustaining progress on neglected tropical diseases [Internet]. 2015 [cited 12 Dec 2017]. Available: http://www.who.int/water_sanitation_health/publications/wash-and-ntd-strategy/en/ 10.1093/inthealth/ihv073 PMC5580794 26940305

