# Supplementary material for: A first nation-wide assessment of soil-transmitted helminthiasis in Fijian primary schools, and factors associated with the infection, using a lymphatic filariasis transmission assessment survey as surveillance platform
Source: PLoS Negl Trop Dis. 2020 Sep 25;14(9):e0008511. doi: 10.1371/journal.pntd.0008511 (PMC7518615; doi:10.1371/journal.pntd.0008511)
Supplement: S2 Table — (DOCX) [file pntd.0008511.s002.docx]

**S2 Table. Distribution of individual, household, and school WASH characteristics of the study participants and STH prevalence in the Western, Central and Northern Divisions of Fiji, 2014-2015**

| **Individual and household characteristics** | **All (n=1,839)** | **Any STH prevalence (%, 95% CI)** | ***Ascaris* infection prevalence (%, 95% CI)** | **Hookworm infection prevalence (%, 95% CI)** |
| --- | --- | --- | --- | --- |
|  |  |  |  |  |
| % Proportion* by current age in years (95% CI) |  |  |  |  |
| 4-6 | 49.6 (46.0-53.3) | 12.4 (7.3-20.4) | 9.6 (4.9-17.9) | 3.6 (1.6-7.0) |
|  | 882/1,751 |  |  |  |
| 7-10 | 50.4 (46.7-54.0) | 11.0 (6.3-18.6) | 7.2 (3.1-15.7) | 4.1 (2.5-6.8) |
|  | 869/1,751 |  |  |  |
| % Proportion* by sex (95% CI) |  |  |  |  |
| Female | 47.9 (44.3-51.6) | 13.0 (7.4-22.0) | 10.2 (4.9-19.8) | 3.2 (1.9-5.5) |
|  | 868/1824 |  |  |  |
| Male | 52.1 (48,4-55.7) | 11.1 (6.8-17.6) | 7.2 (3.6-13.9) | 4.5 (2.4-8.1) |
|  | 956/1,824 |  |  |  |
| % Proportion* by handwashing behavior before eating or after toilet use (95% CI) |  |  |  |  |
| Yes, usually | 66.7 (57.4-74.5) | 7.1 (4.5-10.1) | 3.7 (2.4-5.4) | 4.1 (2.0-8.0) |
|  | 993/1,364 |  |  |  |
| Not always but sometimes | 31.9 (23.8-41.3) | 16.4 (8.6-29.1) | 12.6 (5.7-25.7) | 4.0 (1.8-9.0) |
|  | 354/1,364 |  |  |  |
| Not at all | 1.3 (0.6-3.1) | 30.3 (15.5-50.1) | 12.3 (4.8-28.5) | 17.9 (7.7-36.2) |
|  | 17/1,364 |  |  |  |
| % Proportion* by utensil use during the meals (95% CI) |  |  |  |  |
| Yes, usually | 59.8 (55.8-63.7) | 10.1 (6.0-16.3) | 6.2 (2.8-13.3) | 4.5 (2.5-7.9) |
|  | 797/1,345 |  |  |  |
| Not always but sometimes | 35.3 (30.6-40.4) | 10.3 (6.9-15.3) | 8.7 (5.4-13.5) | 2.1 (0.7-5.7) |
|  | 481/1,345 |  |  |  |
| Not at all | 4.9 (2.7-8.7) | 21.2 (10.2-39.1) | 3.4 (1.1-10.0) | 17.9 (8.1-34.9) |
|  | 67/1,345 |  |  |  |
| % Proportion* by shoes wearing behavior (95% CI) |  |  |  |  |
| Yes, usually | 56.9 (45.4-67.8) | 5.8 (3.4-9.7) | 3.3 (2.2-5.0) | 3.0 (1.2-7.3) |
|  | 882/1,367 |  |  |  |
| Not always but sometimes | 39.6 (29.1-51.2) | 16.2 (10.0-25.1) | 11.7 (6.0-22.4) | 4.7 (1.8-11.3) |
|  | 431/1,367 |  |  |  |
| Not at all | 3.5 (1.9-6.2) | 25.6 (14.7-40.6) | 5.9 (2.2-15.1) | 19.7 (10.0-35.1) |
|  | 54/1,367 |  |  |  |
| % Proportion* who recalls deworming medication ingestion (95% CI) |  |  |  |  |
| Yes | 36.5 (31.1-42.2) | 9.8 (4.9-18.4) | 7.6 (3.6-15.3) | 2.8 (1.2-6.4) |
|  | 494/1,349 |  |  |  |
| No or no answer | 63.5 (57.8-68.9) | 10.4 (6.9-15.2) | 5.8 (3.5-9.5) | 5.0 (2.5-9.7) |
|  | 855/1,349 |  |  |  |
| % Proportion* by main water source at home (95% CI) |  |  |  |  |
| Piped water, Fiji Water Authority | 38.5 (25.2-53.8) | 5.3 (3.1-8.8) | 4.3 (2.5-7.6) | 1.2 (0.4-3.4) |
|  | 709/1,351 |  |  |  |
| Piped water, private or local | 46.5 (33.4-60.0) | 14.6 (8.8-23.3) | 8.4 (3.6-18.4) | 6.8 (4.0-11.5) |
|  | 490/1,351 |  |  |  |
| Others (Rainwater tank, borehole, river or stream) | 15.1 (8.9-24.4) | 11.9 (6.0-22.5) | 8.6 (3.9-18.0) | 3.9 (1.0-13.9) |
|  | 152/1,351 |  |  |  |
| % Proportion* by home latrine type (95% CI) |  |  |  |  |
| Water seal/pour-flush | 85.4 (79.5-89.8) | 9.8 (5.8-16.0) | 6.4 (3.1-12.7) | 3.8 (2.2-6.4) |
|  | 1,212/1,368 |  |  |  |
| Pit latrine | 13.5 (9.1-19.5) | 14.1 (6.7-27.2) | 9.3 (3.6-22.3) | 6.0 (1.9-17.3) |
|  | 134/1,368 |  |  |  |
| River or bush | 1.2 (0.6-2.3) | 26.4 (7.6-61.0) | 7.3 (0.9-40.7) | 19.0 (3.7-58.8) |
|  | 22/1,368 |  |  |  |
| **School characteristics** |  |  |  |  |
| % Proportion* by number of students enrolled in class 1 and 2 |  |  |  |  |
| <75 | 69.8 (53.0-82.6) | 13.6 (7.5-23.4) | 10.7 (4.9-21.8) | 3.1 (2.0-5.0) |
|  | 984/1,839 |  |  |  |
| >75 | 30.2 (17.5-47.0) | 8.5 (3.9-17.5) | 4.0 (2.3-6.8) | 5.5 (1.8-15.5) |
| % Proportion* by number of students attending urban schools | 855/1,839 |  |  |  |
| Yes | 21.8 (11.3-38.0) | 3.9 (1.7-8.7) | 3.5 (1.6-7.7) | 0.6 (0.2-1.8) |
|  | 686/1,839 |  |  |  |
| No | 78.2 (62.0-88.7) | 14.4 (8.5-23.1) | 10.1 (4.8-20.1) | 4.7 (2.7-8.1) |
|  | 1,153/1,839 |  |  |  |
| % Proportion* by main water source at school |  |  |  |  |
| Piped water, Fiji Water Authority | 41.3 (26.8-57.4) | 5.3 (3.1-8.8) | 4.5 (2.5-7.8) | 1.0 (0.5-2.1) |
|  | 1,069/1,834 |  |  |  |
| Piped water, private or local | 28.7 (16.4-45.3) | 16.2 (8.8-28.0) | 9.2 (3.3-23.3) | 8.1 (3.9-16.2) |
|  | 409/1,834 |  |  |  |
| Others (Rainwater tank, borehole, river or stream) | 30.0 (17.4-46.6) | 17.4 (6.7-40.0) | 13.9 (4.0-38.5) | 3.6 (1.6-7.8) |
|  | 356/1,834 |  |  |  |
| % Proportion* by school latrine type |  |  |  |  |
| Water/pour-flush | 97.9 (91.5-99.5) | 12.2 (7.3-19.7) | 8.8 (4.4-16.9) | 3.9 (2.2-6.6) |
|  | 1,806/1,839 |  |  |  |
| River or bush | 2.1 (0.5-8.5) | 6.0 (1.7-18.8) | 3.0 (0.9-9.8) | 3.0 (0.9-9.8) |
|  | 33/1,839 |  |  |  |

This is S2 Table legend.

*****Proportions are weighted based on the proportion of sub-Divisional per Divisional population sizes.
